# Preparation and Characterization of 3D-Printed Biobased Composites Containing Micro- or Nanocrystalline Cellulose

**DOI:** 10.3390/polym14091886

**Published:** 2022-05-05

**Authors:** Raphael Palucci Rosa, Giuseppe Rosace, Rossella Arrigo, Giulio Malucelli

**Affiliations:** 1 Department of Engineering and Applied Sciences, University of Bergamo, Viale Marconi 5, Dalmine, 24044 Bergamo, Italy; 2 Department of Engineering and Applied Sciences, University of Bergamo, Local INSTM Unit, Viale Marconi 5, Dalmine, 24044 Bergamo, Italy; giuseppe.rosace@unibg.it; 3 Department of Applied Science and Technology, Politecnico di Torino, Local INSTM Unit, Viale T. Michel 5, Provincia di Alessandria, 15121 Alessandria, Italy; rossella.arrigo@polito.it (R.A.); giulio.malucelli@polito.it (G.M.)

**Keywords:** acrylated epoxidized soybean oil, poly(ethylene glycol) diacrylate, 3D printing, stereolithography, microcrystalline cellulose, cellulose nanocrystals

## Abstract

Stereolithography (SLA), one of the seven different 3D printing technologies, uses photosensitive resins to create high-resolution parts. Although SLA offers many advantages for medical applications, the lack of biocompatible and biobased resins limits its utilization. Thus, the development of new materials is essential. This work aims at designing, developing, and fully characterizing a bio-resin system (made of poly(ethylene glycol) diacrylate (*PEGDA*) and acrylated epoxidized soybean oil (*AESO*)), filled with micro- or nanocellulose crystals (MCC and CNC), suitable for 3D printing. The unfilled resin system containing 80 wt.% AESO was identified as the best resin mixture, having a biobased content of 68.8%, while ensuring viscosity values suitable for the 3D printing process (>1.5 Pa s). The printed samples showed a 93% swelling decrease in water, as well as increased tensile strength (4.4 ± 0.2 MPa) and elongation at break (25% ± 2.3%). Furthermore, the incorporation of MCC and CNC remarkably increased the tensile strength and Young’s modulus of the cured network, thus indicating a strong reinforcing effect exerted by the fillers. Lastly, the presence of the fillers did not affect the UV-light penetration, and the printed parts showed a high quality, thus proving their potential for precise applications.

## 1. Introduction

Additive manufacture technology (AM), commonly known as 3D printing, allows the creation of complex structures using different types of materials, such as polymers, photopolymer resins, ceramics, and metals [1]. Its freedom of design, low cost, and speed are some of the advantages that make 3D printing attractive for many industrial sectors [2,3], especially in the biomedical field, where it can be used to produce patients’ customized implants [4].

AM can be divided into seven technologies: material extrusion, material jetting, powder bed fusion, direct energy deposition, binder jetting, sheet addition, and vat polymerization, each with its own advantages and limitations [5,6]. Stereolithography, part of the vat polymerization technology, uses a combination of photosensitive liquids made of acrylates or epoxy-acrylates with a photo-initiator and a light emitter (wavelength between 360 and 405 nm) to produce highly accurate components, down to 5 microns of resolution [7,8]. The objects produced by this technique usually exhibit good thermal, mechanical, and chemical properties [9]. However, most of the materials commonly used to produce the resins are derived from crude oil, which, in addition to being nonrenewable and having low biocompatibility, is a major cause of environmental pollution [10].

In recent years, a limited number of academic studies started to address the lack of biocompatible and biobased materials for SLA applications. These new resins are based on renewable materials, such as vegetable oils (soybean, corn, sunflower, linseed, etc.) [11,12,13] or clinically approved polymers such as poly(ethylene glycol) (PEG), poly(caprolactone) (PCL), poly(lactic acid) (PLA), and poly(glycolic acid) (PGA) [14,15,16,17]. Among these different polymers, PEG is one of the best candidates for 3D printing, since it can be easily functionalized and modified [18,19]. Kuo et al. [20] used poly(ethylene glycol)diacrylate (*PEGDA*) with a suitable photo-initiator (namely, Irgacure-819) to create a variety of complex 3D microfluidic devices [20]. Seo et al. [21] employed a similar resin composition to investigate its suitability for complex structures (such as tilted ratchets and ladders) [21]. Unfortunately, the objects produced using pure *PEGDA* and its derivatives via radical polymerization demonstrated poor mechanical properties [22].

An alternative to improve PEG properties while maintaining its biocompatibility could be its combination with other types of resins; in this context, plant-based resins have attractive characteristics due to their renewability, biodegradability, and low pollutant emissions [23]. Originating from renewable sources, they are environmentally friendly molecules, are relatively cheap to manufacture and employ, and can be modified to react with ultraviolet radiation [24,25]. Epoxy-acrylated soybean oil (*AESO*), produced from soybean, has been shown to be a promising material [25]. It was recently used to develop 4D scaffolds [26], complex structures [25], and high-performance parts [27], where it demonstrated good biocompatibility, as well as thermal and mechanical properties. Lebedevaite et al. [28] combined AESO with biobased materials to develop a resin with similar properties to petroleum-based counterparts. Their resins showed high renewable carbon content (between 75% and 82%), high crosslinking rate, and tunable mechanical properties.

Another method to improve the properties of SLA resins is through the addition of fibers, fillers, or nanoparticles [29,30]. These particles can be added directly to the resin or have their chemical structure modified to improve their dispersion and interfacial adhesion, i.e., by attaching a functional group onto the particle surface (“*grafting*”) [31]. Moreover, the functionalization of particles can help in preventing the phase separation between two polymeric components (an A/B mixture) [32]. Among the many types of fillers, cellulose is an excellent reinforcement material. It is the most abundant natural polymer on Earth, and its reinforcing derivatives are environmentally friendly, are relatively cheap to produce, and show impressive mechanical properties [33,34]. Cellulose and its derivatives are also being tested for biomedical applications [35]. Dutta et al. developed a hydrogel containing cellulose nanocrystal (CNC), alginate, and gelatin to be tested for bone regeneration. Their results showed that the scaffolds with CNC had an improvement in cell proliferation when compared to the control group [36]. However, the biomolecular interaction of cellulose micro- and nanoparticles on cells and tissues, as well as their hazard potential, still needs to be further investigated [37]. Nevertheless, for 3D printing, cellulose has already been used to reinforce different types of materials [38,39]. Murphy et al. used microcrystalline cellulose (MCC) to improve the mechanical properties of PLA bio-composites, exploiting its high Young’s modulus (25 ± 4 GPa) [40]. Palaganas et al. successfully combined cellulose nanocrystals with *PEGDA*. Their results showed increased glass transition temperature and tensile strength and decreased swelling when 0.3 wt.% CNCs were incorporated into the diacrylate [15]. 

The main goal of the present work was to develop a high-quality and environmentally friendly biobased system, suitable for 3D printing. To this aim, *PEGDA* was combined with different concentrations of AESO, and their optimum ratio was determined. Then, micro- or nanocrystalline cellulose was incorporated into the optimized *PEGDA*/*AESO* formulation, and Fourier-transform infrared spectroscopy (FTIR) was exploited for monitoring the progress of the UV curing of the designed system during the SLA process. Furthermore, the rheological, thermal, mechanical, and 3D printing properties of the biobased composites were investigated. To the best of our knowledge, this is the first time that *PEGDA*, *AESO*, MCCs, and CNCs have been combined and tested for 3D-printing applications. The obtained results show the possible exploitation of cellulose-reinforced biobased materials in the 3D printing industry.

## 2. Materials and Methods

### 2.1. Materials

Poly(ethylene glycol) diacrylate (number average molecular weight: 575), epoxy-acrylate soybean oil (containing 4000 ppm of monomethyl ether hydroquinone as inhibitor), diphenyl(2,4,6-trimethylbenzoyl)phosphine oxide (TPO), and cellulose microcrystalline powder (20 μm) were purchased from Merck KGaA (Darmstadt, Germany). Cellulose nanocrystals were purchased from Sappi limited (Maastricht, the Netherlands). Isopropanol (purity: 99%) was purchased from Carlo Erba (Cornaredo, Italy). All products were used as received.

### 2.2. Preparation of the Photocurable Systems

**Scheme 1:**
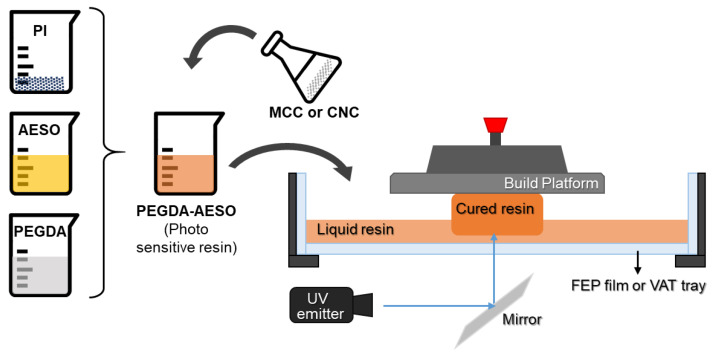
The 3D printing process for the photocurable systems.

Photocurable resins were prepared while avoiding any exposure to light, and the procedure is presented in Scheme 1. First, using a lightproof beaker, different weight ratios (varying from 50 to 90 wt.%) of *AESO* were mixed with poly(ethylene glycol). Then, 2 wt.% TPO was added to the mixtures, which were ultrasonicated for 10 min at room temperature to dissolve the photo-initiator, before vigorously stirring for 30 min. The resulting mixture had a yellowish appearance.

The bio content (*BC*) of each *PEGDA*/*AESO* sample is described in Table 1 and was calculated according to Equation (1).

(1) $$BC=w_{i}\times BRC_{PEGDA}+w_{i}\times BRC_{AESO},$$

where *w* is the mass fraction, *i* is the resin type, and bio-renewable carbon (*BRC*) is the ratio of bio-sourced carbon to the sum of bio- and fossil-based carbons. The *BRC* values of *AESO* and *PEGDA* are 86% and 0%, respectively [41].

The mixture of *PEGDA*/*AESO* with a 20:80 ratio was selected for the present investigation since it had the best mechanical properties with the most significant bio-content percentage, equal to 68.8%. Then, different percentages of MCCs or CNCs (ranging from 0.15 to 2.4 wt.%) were incorporated into the selected mixture. The fillers were added in a small amount (maximum 0.2 g at a time) and ultrasonicated for 10 min at 30 °C to avoid the formation of aggregates.

### 2.3. 3D Printing Process

The samples were printed using a Peopoly moai 130 SLA 3D printer with an easy-to-level build plate. The machine was equipped with a solid-state laser with frequency conversion, emitting at 405 nm with a power of 150 mW. The power level was 58, and the initial exposure time was in the range between 40 and 60 s. The printed process was carried out maintaining the temperature constant at 25 °C. After the end of the printing, the excess of resin was drained and washed off with isopropanol; then, the samples were gently dipped into distilled water for 5 min. Finally, the samples were dried using a paper towel and transferred to a UV chamber equipped with a 405 nm UV lamp to post-cure for 40 min. The post-curing time was the same for all samples to ensure a complete photopolymerization, as assessed by FTIR spectroscopy. The 3D models were created using FreeCad software, and the printing layer height of the samples was set at 0.1 mm.

### 2.4. FTIR Spectroscopy

Fourier-transform infrared spectroscopy (FTIR) was used to confirm the formation of *PEGDA*:*AESO* copolymers at different ratios and to monitor the conversion of the acrylic double bonds. The infrared analysis was performed using a Thermo Avatar 370 spectrophotometer equipped with an attenuated total reflectance (ATR) device with a diamond crystal for solid analysis. Using Omnic 7.3 software, the spectra were collected in absorbance mode with a resolution of 4 cm^−1^ and 32 scans per measurement, within the range of 2000 to 650 cm^−1^, using 3D-printed dog-bone specimens. The conversion of double bonds was calculated from the decrease in the area of the double-bond absorption peak at 810 cm^−1^, as shown by Equation (2).

(2) $$\mathrm{Conversion}\;\left(\%\right)=\left(1-\frac{A_{t\left(810\right)}}{A_{0\left(810\right)}}\right)\times 100\%,$$

where A_0(810)_ and A_t(810)_ are the areas of the peak at 810 cm^−1^, before and after exposure to UV irradiation, respectively.

### 2.5. Rheological Analysis

Rheological measurements were performed using an ARES (TA Instrument, Waters LLC, New Castle, DE, USA) strain-controlled rheometer in parallel plate geometry (plate diameter: 50 mm; gap between the plates: 0.7 mm). The complex viscosity of the samples was measured through strain sweep measurements in a range of strain amplitude from 1% to 400%. In all tests, the frequency was fixed at 1 rad·s^−1^. All measurements were performed at room temperature.

### 2.6. Tensile Measurements

All tensile tests were performed following the ASTM D638 standard. Five samples were printed with a dog-bone shape with the dimensions of 63.5 mm × 9.53 mm × 3.2 mm (L × W × T) and with 3.2 mm width in the narrow section. The tests were performed at room temperature with an Instron 5966 dynamometer (Norwood, MA, USA), equipped with 5 kN load cell. Each test was performed at a crosshead speed of 10 mm/min. Tensile strength (MPa) and elongation at break (%) were determined using the average of the five tests. Then, various mechanical properties were calculated using the data from the tensile curves, such as Young’s modulus (MPa) and fracture energy (mJ).

### 2.7. Scanning Electron Microscopy

The morphology of the obtained 3D-printed systems was studied using an EVO 15 scanning electron microscope (SEM) from Zeiss (Oberkochen, Germany), coupled with an Ultim Max 40 energy dispersive X-ray (EDX) microanalyzer by Oxford Instruments (High Wycombe, UK); the samples were fractured in liquid nitrogen, fastened to a conductive adhesive tape, and finally gold-metallized. The samples were analyzed using a secondary electron detector, with energy set at 20.00 kV, at two magnifications (1000× and 2500×).

### 2.8. Thermogravimetric Analysis

The thermal and thermo-oxidative stability of all prepared systems was assessed by thermogravimetric (TG) analyses carried out on a Discovery apparatus (TA Instruments), from 50 to 700 °C, using a heating rate of 10 °C·min^−1^, under both nitrogen and air flow (35 and 25 mL·min^−1^, respectively). The experimental error was ±0.5% for the weight and ±1 °C for the temperature.

### 2.9. Swelling Behavior

Five rectangular samples with dimensions of 30 mm × 10 mm × 5 mm (L × W × H) were prepared with different *PEGDA*:*AESO* weight ratios and CNC or MCC loadings and then weighed (*M*_1_). The samples were then dipped into deionized water for a total period of 30 days. Every 5 days, the samples were taken from the water, dried using a dry cloth, and weighed again (*M*_2_). The swelling ratio, *S_w_*, was calculated using Equation (3).

(3) $$S_{w}=\frac{\left(M_{2}-M_{1}\right)}{M_{1}}\times 100\%.$$

### 2.10. Contact Angle Measurement

Static contact angle values of *AESO*/*PEGDA* samples were measured using a homemade instrument equipped with a high-speed CCD camera. The used equipment allows the determination of contact angle, with a precision of ±1°, by taking images at frequencies as high as 200 Hz, starting within a few tens of milliseconds after the deposition of the drop. All measurements were performed at room temperature and relative humidity RH = 40% ± 5%. Five rectangular-shaped parts (size: 50 mm × 25 mm × 1 mm) were 3D-printed for each formulation. A small drop of high-purity distilled water was slowly placed on the surface of the samples, and various photos of the droplet were recorded after 5 s. The volume of the water drop was 4 ± 0.5 μL. The contact angle (CA) values were determined using image analysis software. 

### 2.11. Working Curve

**Figure 1:**
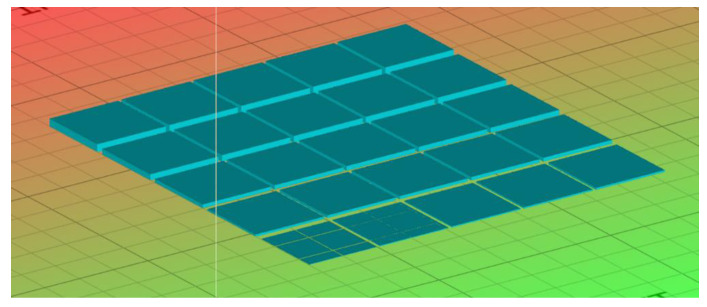
The 3D model used to determine the resin curing characteristics.

Since the kinetics during the curing process is highly complex, simplified equations are commonly used to describe the kinetics during photopolymerization [42]. In this study, an equation was adapted from the Beer–Lambert relationship, expressing the exponential decay of the light intensity as it passes through an absorbing medium i [43,44]. This semiempirical equation relates the thickness of the crosslinked layer (cure depth) to the irradiation dose through the following equation:

(4) $$C_{d}=D_{p}\ln\left(\frac{E_{max}}{E_{c}}\right),$$

where *C_d_* is the cure depth of a single layer, *D_p_* is the penetration depth at which the intensity of the beam is reduced to 1/e^2^ (13.5%) of its value at the surface, *E_c_* is the exposure per unit of area needed for the resin to reach its gel point, and *E_max_* is the laser exposure on the resin surface. *D_p_* and E_c_ were estimated by varying the resin exposure and measuring the cured layer depth. By plotting *C_d_* vs. ln(*E_max_*), a line known as the working curve was obtained. *D_p_* was calculated from the slope of the line, and *E_c_* was the X-intercept. To this aim, a square part composed of 25 equal-area square tiles (1 mm^2^), based on the model described by Bassett et al. [45], as shown in Figure 1, was printed using the Peopoly moai 130 SLA printer.

The part had 25 layers in total, with the height of each layer being 0.05 mm. Each square was exposed to light in an arithmetic progression increasing from 1 to 25. Furthermore, to avoid the effect of light scattering, the squares were separated by a gap of 1 mm. The height of each tile was measured three times using a digital caliper with 0.01 mm of resolution (Preciva Digital Caliper) and a ±10% deviation was assumed [46]. Finally, with the height of each tile, the maximum exposure of a tile was calculated using Equation (5).

(5) $$E_{max}=\sqrt{\frac{2nP_{L}}{\pi W_{0}V_{s}}},$$

where *n* is the number of times the laser passed over the tile, *P_L_* is the laser power, *W*_0_ is the beam width, and *V_s_* is the scanning speed. *P_L_* and *W*_0_ values are specific to the 3D printer; in this case, they were 150 mW and 0.07 mm, respectively. Three parts were printed with scanning speeds of 80 mm·s^−1^ and 200 mm·s^−1^.

When the MCCs and CNCs were added to the mixture, the light scattering effect exerted by these particles needed to be taken into account [44]. Thus, *D_p_* was adjusted to consider the scattering effect, as shown by Equation (6).

(6) $$D_{p}=\frac{2}{3}\frac{d}{Q\phi },$$

where *d* is the filler mean size, *φ* is the filler volume fraction, and *Q* is the scattering efficiency. *Q* was calculated according to Equation (7), in which *λ* is the irradiation wavelength, *h* is the interparticle distance, and Δ*n* is the difference in refractive index between the filler (*n_f_*) and the photosensitive material (*n*_0_).

(7) $$Q=\frac{h}{\lambda }\Delta n^{2}.$$

## 3. Results

### 3.1. Biobased Resin Characterization

#### 3.1.1. FTIR Analysis

**Figure 2:**
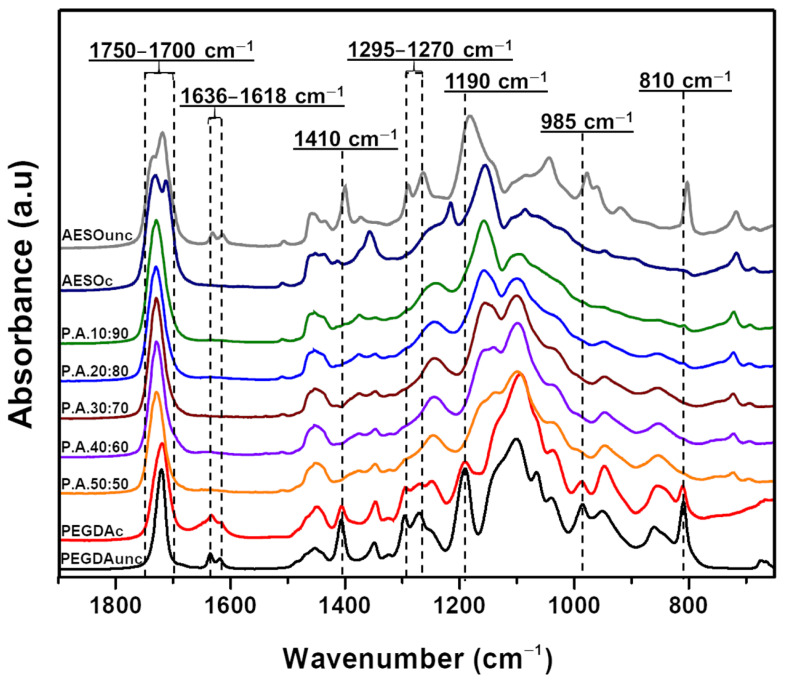
FTIR spectra of *PEGDA* mixed with different *AESO* loadings (c = cured; unc = uncured).

The FTIR spectra of 3D-printed specimens obtained by uncured and UV-cured *AESO*, *PEGDA*, and their combinations are shown in Figure 2. In the wavenumber domain between 1800 and 700 cm^−1^, the infrared spectrum of uncured AESO showed peaks between 1750 and 1700 cm^−1^ and at 1270 cm^−1^ attributed to carbonyl and ester groups, respectively. In particular, the presence of two different absorbance bands at 1737 and 1723 cm^−1^, both assigned to C=O, is consistent with a different chemical environment for carbonyl groups on monomers. Furthermore, four characteristic absorption peaks were assigned to the stretching vibration of the C=C double bonds at 1636, 1618, 1410, and 810 cm^−1^ [47,48]. Further infrared absorption bands for *PEGDA* were assigned to the C–O bonds of the ester groups (1270 cm^−1^, 1190 cm^−1^, and 985 cm^−1^) [49,50]. After the curing process, the FTIR spectra of both resins showed significant changes due to irradiation and copolymerization. In fact, under UV irradiation, the photo-initiator produced active radicals that opened the double bonds in the monomers, thereby promoting crosslinking reactions. The occurrence of photopolymerization was verified by monitoring the double-bond peaks at 1636, 1618, 1410, and 810 cm^−1^ [11,51,52,53,54,55]. As shown in Figure 2, at the end of the UV curing step, for the *PEGDA* sample, the double-bond peaks were reduced drastically, which means that they were continuously combining with the created radicals, participating in curing and increasing the crosslinking density. These modifications were more evident in the case of *AESO* resin, for which the disappearance of the infrared absorption bands assigned to unsaturated C=C bonds indicated that acrylate groups were completely depleted during the UV curing process. The absence of infrared bands assignable to unsaturated C=C bonds in *PEGDA*–*AESO* spectra suggests the successful curing of *PEGDA*/*AESO* formulations. More specifically, the presence of *AESO* in the 3D printing formulations accounted for a higher conversion of *PEGDA* acrylic groups when *PEGDA* content was below 20 wt.%, as confirmed by the small peak observed at 810 cm^−1^ only for the samples containing 80 and 90 wt.% *AESO* [56]. Under the employed curing conditions, the presence of *AESO*, between 30% and 50%, significantly increased the conversion of C=C bonds measured by the area of the peak at 810 cm^−1^, reaching 100% for some of the investigated formulations (see Table 2). This phenomenon could be attributed to the structure of AESO, which has many polar groups (i.e., hydroxyl and epoxy groups) capable of interacting with *PEGDA* [57]. This result is particularly interesting for biomedical applications. In fact, the conversion of double bonds is directly related to the material biocompatibility issues, as the presence of unreacted free-radical groups may cause irritation and damage to the soft tissue [58].

#### 3.1.2. Rheological Behavior

During the 3D printing process, each time the build platform is raised, a gap is formed between the polymerized layer and the VAT containing the resin. The resin must be fluid enough to be able to fill this gap [30]. Thus, controlling the viscosity is essential when developing a resin for 3D printing applications. The viscosity of commercial resins, such as Formlabs or Anycubic, is usually between 0.1 Pa·s and 1.5 Pa·s [45]. The viscosity of pure *PEGDA* is 0.057 Pa·s, while pure *AESO* has a viscosity of 15 Pa·s (which is too high for 3D printing). When 50 to 90 wt.% *AESO* was mixed with *PEGDA*, the resin viscosity increased according to the *AESO* loading, from 0.057 (pure *PEGDA*) to 0.38 (*AESO* loading: 50 wt.%), 0.25 (60 wt.%), 0.80 (70 wt.%), 1.27 (80 wt.%), and 2.85 (90 wt.%) Pa·s. The mixture containing 90 wt.% *AESO* was too viscous for the machine to print at room temperature, resulting into failed parts. Therefore, 80 wt.% was the maximum amount of AESO that could be mixed with *PEGDA*.

#### 3.1.3. Mechanical Properties

**Figure 3:**
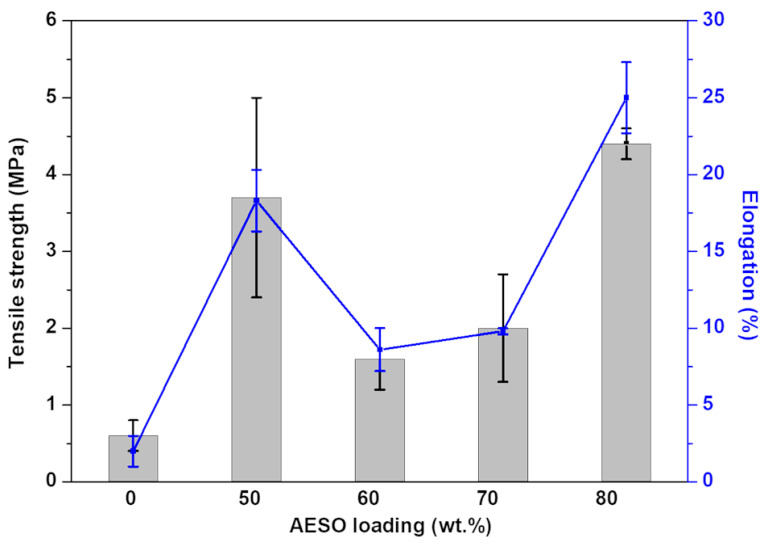
Variation of the tensile strength and elongation as a function of *AESO* loading. Statistical analysis for the elongation at break was performed using a one-way analysis of variance (ANOVA) with the level of significance set at probabilities of *p* < 0.05.

The capability to tune and control the mechanical properties of 3D-printed systems is very important when developing objects for biomedical applications [10]. In the case of biomaterials, their molecular characteristics have a strong influence on the overall mechanical behavior [25,59]. The tensile strength of pure *PEGDA* is approximately 0.6 MPa ± 0.2 MPa [15,60]. When *PEGDA* was combined with *AESO*, the tensile strength showed a general increase (Figure 3) by 633% (4.4 ± 0.2 MPa) when 80 wt.% *AESO* was added to the 3D printing formulation. However, at the same time, the samples containing 60 and 70 wt.% of *AESO* demonstrated a lower tensile strength gain when compared with the other samples. This effect could be an indicative of network loosening [24], requiring a longer post-curing time.

The elongation at break is related to the material’s capability to bend and deform to some extent without cracking [61]. Usually, plant-based resins have a high elongation at break due to the presence of free fatty acids in their network structure [24,25,28]. The addition of *AESO* resulted in an increase in elongation for all samples (Figure 3). Pure *PEGDA* achieves a maximum elongation of 2% ± 1% [15]. When 50 and 80 wt.% *AESO* were added, the elongation at break increased by 815% (18.3% ± 2.0%) and 1150% (25% ± 2.3%), respectively. 

As shown in Figure 3, among all samples, the formulation containing 80 wt.% *AESO* provided the best mechanical properties while having the highest bio-content percentage, equal to 68.8% (Equation (1)). Thus, it was selected as the base formulation for the incorporation of MCCs and CNCs.

### 3.2. Effect of the Incorporation of Micro- and Nanocrystalline Cellulose in 3D Printing Formulations

#### 3.2.1. UV Curing

FTIR was also used to investigate the impact of the two fillers on the UV curing process. As shown in Table 3, the incorporation of MCCs and CNCs into the resin system containing 80 wt.% *AESO* did not have a significant impact on the conversion. The spectra, which displayed the typical bands of cellulose, are shown for both fillers in Figure S1. The broad peaks at around 3400–3300 cm^−1^, 2918–2849 cm^−1^, and 1430–1428 cm^−1^ were assigned to O–H stretching, to the asymmetric and symmetric stretching of methylene (–CH_2_–) groups in long alkyl chains, and to C–H in-plane bending of cellulose, respectively. Additionally, the infrared absorption bands at 1369, 1316, 1053 and 897 cm^−1^ were ascribed to C–H deformation stretching, C–H wagging and in-plane ring stretching, C–O stretching, and C–O–C stretching of the β-(1→4)-glycosidic linkage in cellulose, respectively. The incorporation of MCC and CNC into the *PEGDA*/*AESO* formulation was not expected to affect the UV-curing process; in fact, the same bands were still visible in the spectrum of the composite containing 2.4 wt.% MCCs or CNCs, where the signals at 1630 cm^−1^ and 806 cm^−1^, associated with stretching vibration for asymmetric vibration of out-of-plane vinyl groups, indicated the presence of unreacted double bonds.

#### 3.2.2. Rheological Behavior

The addition of fillers can also increase the resin viscosity; thus, their form, sizes, and properties have a great impact on the 3D-printed outcome [62]. In order to test the influence of MCCs and CNCs in the optimized resin formulation, different amounts of fillers were incorporated. As shown in Table 4, both MCCs and CNCs showed a similar effect, irrespective of the size. However, the resin viscosity was slightly higher when MCCs were dispersed into the UV-curable system. This difference could be attributed either to the larger particle size of MCCs or to the formation of small aggregates, as shown by SEM analyses (see Section 3.2.4).

#### 3.2.3. Mechanical Behavior

**Figure 4:**
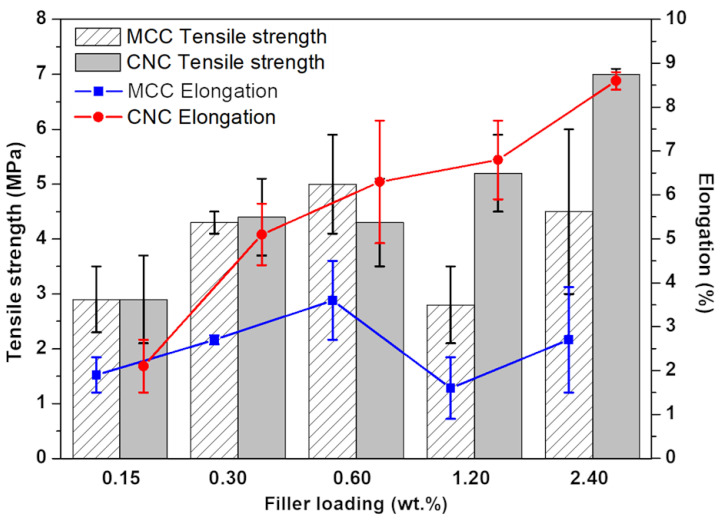
Influence of MCC and CNC loadings on P.A.20:80 tensile strength and elongation at break. Statistical analysis for the elongation at break was performed using a one-way analysis of variance (ANOVA) with the level of significance set at probabilities of *p* < 0.05.

The addition of MCCs and CNCs to the P.A.20:80 mixture had opposite effects on tensile strength and elongation (Figure 4). When the fillers were incorporated into P.A.20:80, the elongation at break drastically decreased, from 25% ± 2.3% (unfilled resin system) to 3.6% ± 0.9%, with 0.6 wt.% MCC, and to 8.6% ± 0.2%, with 2.4 wt.% CNCs (i.e., corresponding to the highest loading). On the other hand, the tensile strength increased by 2.3% and 59.1% when 2.4 wt.% MCCs or CNCs were added, respectively. These findings can be attributed to the inherent stiffness of the cellulose structure [63]. However, although both fillers are derived from cellulose, CNCs performed better at higher loadings than MCCs; this finding could be ascribed to (i) the lower ductility of MCCs that may result in early breaks, or (ii) the formation of aggregates, which can decrease the tensile strength, as already observed by dos Santos et al., who incorporated CNCs and MCCs into poly(lactide) [64].

**Figure 5:**
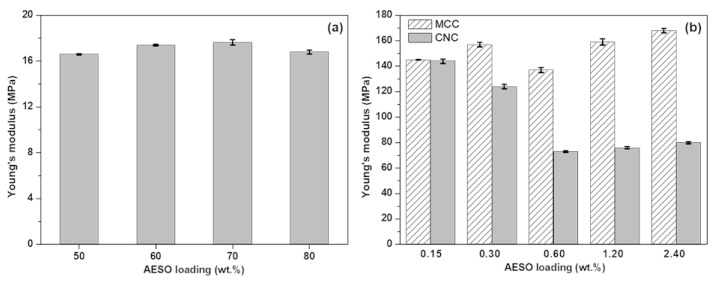
Young’s modulus values of *PEGDA*/*AESO* 3D-printed systems (**a**) and of P.A.20:80 containing different amounts of MCCs and CNCs (**b**).

The Young’s modulus is generally used for assessing the stiffness of a solid material [65]. Unfilled *PEGDA* is reported to have a Young’s modulus of 26 ± 1 MPa [15]. However, when different concentrations of *AESO* were added, Young’s modulus decreased on average by 34.7%, as shown in Figure 5a. Furthermore, changing the *AESO* load did not affect the Young’s modulus; this finding may indicate that the elastic deformation was mainly dictated by *AESO*–*AESO* interaction.

The incorporation of MCCs and CNCs remarkably increased the Young’s modulus of the optimized resin formulation. As observed in Figure 5b, when 0.15 wt.% MCCs or CNCs were incorporated, both fillers determined an increase in Young’s modulus from 16.8 ± 0.17 MPa to 145 ± 0.16 MPa and 144 ± 1.74 MPa, respectively. However, as the weight percentage of fillers in the resin system increased, the presence of MCCs resulted a higher Young’s modulus, nearly achieving two times the value observed in the presence of CNCs, when 2.4 wt.% of each filler was used. This variation could be due to the differences in morphology and chemical surface structure of the fillers, as already reported in the scientific literature [64,66].

#### 3.2.4. Scanning Electron Microscopy

**Figure 6:**
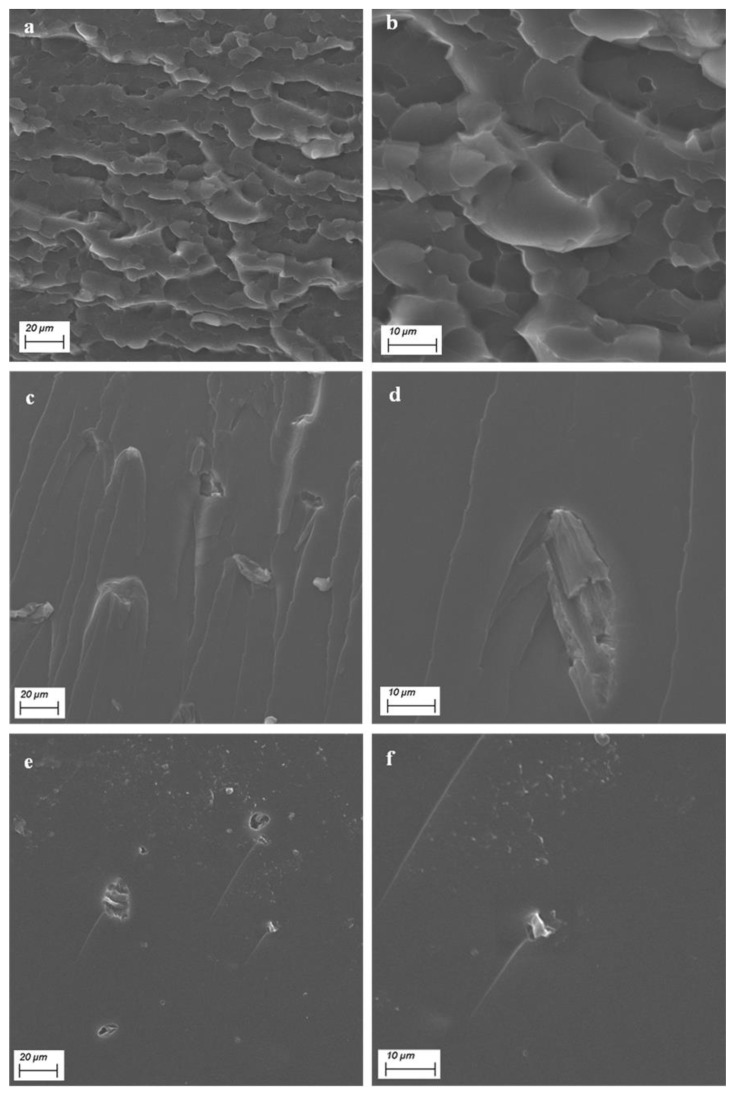
Typical SEM micrographs of (**a**,**b**) unfilled P.A.20:80 and samples containing 0.60 wt.% (**c**,**d**) MCC and (**e**,**f**) CNC.

SEM analysis was carried out to assess the morphology of the composites and the level of distribution of the micro- and nanocellulose crystals. Some typical SEM images are shown in Figure 6. Unfilled 3D-printed P.A.20:80 (Figure 6a,b) showed a rough surface, which became quite smooth when each filler was embedded. The micrographs of the systems containing MCCs and CNCs ((Figure 6c–f), respectively) showed the achievement of good interfacial adhesion between the embedded fillers and the resin. Furthermore, it is noteworthy that both the systems were prone to forming some aggregates (average size between 10 and 20 microns), although the overall dispersion was quite uniform.

#### 3.2.5. Thermogravimetric Analysis

Thermogravimetric analyses were carried out to assess the thermal and thermo-oxidative behavior of the different UV-cured systems. Table 5 presents the obtained data for the 3D-printed unfilled systems investigated as a function of *AESO* content; Table 6 and Table 7 show the results for the 3D-printed composites (resin system: P.A.20:80) containing MCCs and CNCs, respectively.

In nitrogen, the degradation of both *PEGDA* and *AESO*, as well as of their UV-cured mixtures, took place with a single degradation step between 300 °C and 500 °C, during which a progressive breaking of the (co)polymer network occurred. Conversely, the degradation in air showed two steps; the first involved the breaking of the (co)polymer network (between about 380 °C and 480 °C), and the second referred to the oxidation of the products formed during the first step. As observed from the data collected in Table 5, increasing the acrylated epoxidized soybean oil content decreased the T_5%_ values in either air or inert atmosphere. This finding can be ascribed to the chemical structure and composition of *AESO*, which made the molecule less stable and, hence, more prone to degradation; meanwhile, the bio-sourced resin showed a sort of charring effect, as witnessed by the increase in residues collected in nitrogen at the end of the test.

**Figure 7:**
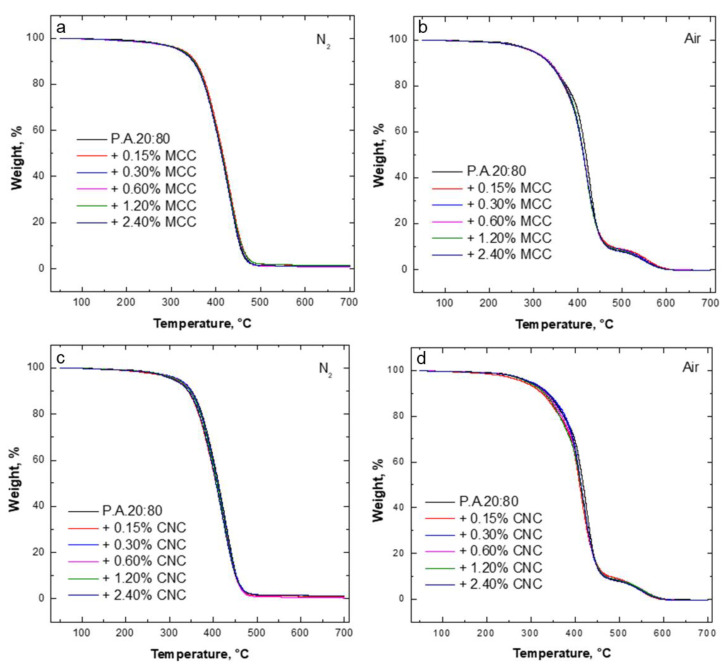
Thermogravimetric curves of the resin mixture containing 80 wt.% *AESO* with varying concentrations of MCC (**a**,**b**) and CNC (**c**,**d**) in air and N_2_ heated at a constant rate of 10 °C·min^−1^.

Furthermore, as shown in Figure 7, the incorporation of micro-cellulose crystals into the optimized resin mixture containing 80 wt.% *AESO* seemed to have a very limited effect on the thermal and thermo-oxidative stability of the 3D-printed composites; in fact, all the characteristic temperatures were almost unchanged and very close to those of the unfilled system (see Table 6). A similar behavior was also found for the 3D-printed composites containing different amounts of nanocellulose crystals (see Table 7), notwithstanding a slight decrease in the T_5%_ values in nitrogen for all filled systems, regardless of CNC loading.

#### 3.2.6. Swelling Properties

**Figure 8:**
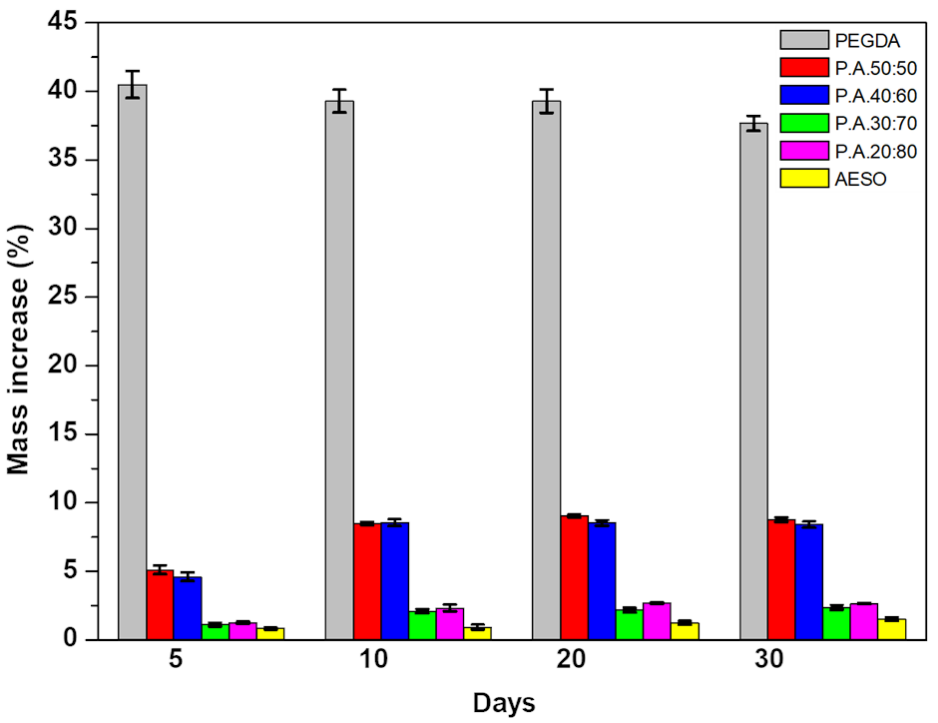
Swelling behavior of 3D-printed parts for 5, 10, 20, and 30 days with different *AESO* loadings.

The ability to not deform in an aqueous solution is crucial for tissue engineering when designing implants [15,18]. Changes in the material’s volume can result in deformations, such as wrinkles and surface breaks, which affect the long-term mechanical resistance of the component [67,68]. As shown in Figure 8, pure *PEGDA* absorbed between 38% and 40% of its weight in water over a 30 day period. The addition of *AESO* drastically decreased the water sorption of the 3D-printed specimens. After 10 days of immersion in water, the water sorption of the samples containing *AESO* stabilized without further significant changes. The samples containing 50 and 80 wt.% *AESO* showed swelling values of 9.4% (a 76% decrease) and 2.75% (a 93% decrease), respectively, after 30 days. The swelling decrease could be attributed to two factors: the *AESO* large carboxylic chains that prevented water molecules from interacting with the *PEGDA* hydroxyl groups and the increase in crosslinking density [69]. In fact, as more *AESO* was added to *PEGDA*, the chains became more entangled (and compact), further decreasing the space into which water could diffuse.

When MCCs and CNCs were added to P.A.20:80, only a small variation in swelling was observed after 30 days of immersion; moreover, swelling values were in the range between 2% and 2.5% (Figures S3 and S4). This means that the addition of the fillers did not significantly impact the water sorption of the 3D-printed parts. Nevertheless, increasing the concentrations of MCC and CNC in the mixture led to higher swelling. This finding could be attributed to the high number of –OH groups present in the cellulose structure, which increased the hydrophilicity [60]. Lastly, all the printed parts showed good mechanical integrity during the 30 days of immersion in water.

#### 3.2.7. Contact Angle

In general, surfaces with moderate wettability are more able to bind to cells and tissues as compared with highly hydrophobic or hydrophilic surfaces [15,70,71]. Therefore, to determine the surface wettability of investigated composites, contact angles of *PEGDA* and *AESO*, as well as their combinations, were evaluated. *PEGDA*, which is known to be hydrophilic [72], showed a contact angle of 72.1° ± 3.83°, similar to what has been reported in the literature [15]. On the other hand, *AESO* displayed a contact angle of 92° ± 1.6°, indicating that it is practically hydrophobic. In fact, even though AESO bears some hydroxyl and epoxy functionalities (i.e., hydrophilic groups), it mainly consists of large nonpolar carboxylic chains [57]. Thus, upon adding *AESO*, the *PEGDA* wettability decreased (Table S1); the P.A.20:80 mixture exhibited a contact angle of 93° ± 2.3°, i.e., very close to that of pure *AESO*.

When MCCs and CNCs were dispersed in P.A.20:80, its wettability increased (see Table 8). This finding can be attributed to the high number of –OH groups on the surface of the cellulose, which enhanced the sample interaction with water. The contact angle decreased by about 34%, in the presence of MCCs, and by 43%, in the presence of CNCs. However, the wettability increase was not dependent on the filler concentration.

#### 3.2.8. Working Curve Parameters

During the development of a photosensitive resin, it is important to know the correct amount of light or time needed to perform the curing. Models containing many empty spaces in their structure, being overexposed during the printing process, can result in these spaces also being cured. On the other hand, if the resin is underexposed, smaller structures could show deformations or fail to be printed [73]. A good rule of thumb is that the SLA resin should exhibit low values of E_c_ and high values of *D_p_*, as it will need lower energy doses and the radiation will penetrate deeper in the resin [74].

The working curve parameters for the P.A.20:80 resin, *D_p_* and *E_c_* (Equation (4)), were calculated by ordinary least squares (OLS) regression of the cure depth *C_d_* vs. ln(*E_max_*). The regression had an *R*^2^ of 0.98. *E_c_* was equal to 1.09 mJ·mm^−2^, which is in the range of other commercial resin formulations. On the other hand, the resin *D_p_* value was 0.43 mm, which is higher than other commercial resins, such as PR48 (0.053 to 0.105 mm) and VeroWhitePlus (0.145 mm) [46,75]. Nevertheless, if needed, P.A.20:80 can be combined with photo-blocking agents to reduce its sensitivity to light.

Fillers can also reduce the resin sensitivity to light; this depends on the material type and loading. Usually, when fillers are incorporated into the polymer matrix, they tend to scatter the light, blocking part of the UV radiation reacting with the photo-initiators [76]. As shown in Table 9, as the concentration of MCC and CNC particles increased, *D_p_* and *E_c_* decreased. The lowest value of *D_p_* was reached when 2.4 wt.% cellulose was incorporated; specifically, it shifted from 0.43 mm to 0.32 mm, with MCC, and to 0.35 mm, with CNC. Although this decrease would imply that more energy (or time) would be needed to perform the UV curing, its value is still higher than other standard commercial resins. Similarly to *D_p_*, *E_c_* also decreased as the filler content increased, as the fraction of liquid resin needing to be solidified was lower. Lastly, the difference between MCC and CNC *E_c_* values could be related to their particles sizes [76].

## 4. Discussion

The development of biobased resins for 3D printing is essential to reduce the impact of fossil-based materials in nature and to design new applications. This study focused on the development and reinforcement of a biocompatible system capable of printing complex structures. The resin system was first optimized in order to identify the best *PEGDA*-to-*AESO* ratio suitable for the 3D printing process, i.e., with an appropriate viscosity, good mechanical properties, high bio-content, and high reactivity; in particular, the mixture containing 80 wt.% *AESO* was the most performing and was, therefore, chosen as the reference matrix for dispersing micro- and nanocellulose crystals, assessing their potential as fillers for 3D-printed composites. The incorporation of the fillers at different loadings (up to 2.4 wt.%) into the resin formulation containing 80 wt.% *AESO* did not interfere with the 3D printing process, nor had a significant impact on the resin critical energy and depth penetration. Furthermore, the presence of the cellulose crystals, irrespective of their size, increased the wettability of the printed parts (with a lowering of the water contact angle values by about 34% and 43%, when 2.4 wt.% CNCs and MCCs were incorporated, respectively). This finding was attributed to the highly polar characteristics of the fillers, bearing several hydroxyl functionalities. Meanwhile, as assessed by mechanical tests, the tensile strength increased by about 59%, in the presence of 2.4 wt.% CNCs, and the elastic modulus increased by 890%, when the same MCC loading was employed. However, further research on the influence of *AESO*:*PEGDA* resin and its combination with CNCs and MCCs on cellular proliferation and tissue response is needed to better understand its impact on living cells. In conclusion, the composite system developed in this work may represent a sustainable solution to the increasing demand for new environmentally friendly materials for additive manufacturing processes.

## Figures and Tables

**Table 1:** Sample code, composition, and bio-content (*BC*) of each *PEGDA*/*AESO* mixture.

Sample Code	*PEGDA* (%)	*AESO* (%)	*BC* (%)
P.A.50:50	50	50	43.0
P.A.40:60	40	60	51.6
P.A.30:70	30	70	60.2
P.A.20:80	20	80	68.8
P.A.10:90	10	90	77.4

**Table 2:** Double-bond conversion at 810 cm^−1^ for *PEGDA*, *AESO*, and their mixtures.

Sample Code	Peak Area at Initial Time (cm^2^)	Peak Area after 3D Printing (cm^2^)	Conversion
(%)			
*PEGDA*	303.47	84.72	72.0
P.A.50:50	227.91	0	100
P.A.40:60	200.15	0	100
P.A.30:70	204.45	0	100
P.A.20:80	189.90	2.39	98.7
P.A.10:90	180.49	8.76	95.1
*AESO*	161.04	4.94	96.9

**Table 3:** Double-bond conversion at 810 cm^−1^ in the presence of different loadings of MCCs and CNCs in P.A.20:80.

Filler Type	Filler Percentage (%)	Peak Area at Initial Time (cm^2^)	Peak Area after 3D Printing (cm^2^)	Conversion (%)
MCC	0.15	195.23	8.36	95.7
	0.30	200.92	7.56	96.2
	0.60	200.54	0.17	99.9
	1.20	200.73	1.84	99.1
	2.40	186.96	7.77	95.8
CNC	0.15	175.08	2.17	98.8
	0.30	173.32	5.29	96.9
	0.60	177.20	1.08	99.4
	1.20	173.34	7.78	95.5
	2.40	187.90	7.12	96.2

**Table 4:** Complex viscosity values of the UV-curable systems as a function of the filler loading in P.A.20:80.

Filler Type	Loading (wt.%)	Complex Viscosity (Pa·s)
-	-	1.27
MCC	0.15	1.77
	0.30	1.92
	0.60	2.01
	1.20	2.11
	2.40	2.20
CNC	0.15	1.62
	0.30	1.75
	0.60	1.86
	1.20	1.96
	2.40	2.05

**Table 5:** Thermogravimetric data for the different *AESO*/*PEGDA* 3D-printed systems (*w*/*w*).

Sample Code	Atmosphere	T_5%_ (°C)	T_max1_^a^ (°C)	Residue at T_max1_ (%)	T_max2_^a^ (°C)	Residue at T_max2_ (%)	Residue at 700 °C (%)
*PEGDA*	Nitrogen	337	420	37.9	-	-	0.5
P.A.50:50		339	429	38.9	-	-	0.5
P.A.40:60		336	429	36.9	-	-	0.9
P.A.30:70		332	432	33.9	-	-	1.0
P.A.20:80		322	428	33.1	-	-	1.4
*AESO*		308	390	59.0	-	-	2.7
*PEGDA*	Air	308	413	37.2	538	2.4	0
P.A.50:50		310	424	43.6	554	2.6	0
P.A.40:60		312	428	38.8	528	4.0	0
P.A.30:70		308	425	39.1	539	3.6	0
P.A.20:80		299	426	39.5	554	3.7	0
*AESO*		293	397	54.0	552	5.7	0

^a^ From derivative curve.

**Table 6:** Thermogravimetric data for MCC-based composites.

Sample Code	Atmosphere	T_5%_ (°C)	T_max1_^a^ (°C)	Residue at T_max1_ (%)	T_max2_^a^ (°C)	Residue at T_max2_ (%)	Residue at 700 °C (%)
P.A.20:80	Nitrogen	322	428	33.1	-	-	0.9
+0.15% MCC		322	431	34.2	-	-	1.1
+0.30% MCC		320	430	32.1	-	-	0.9
+0.60% MCC		317	429	32.9	-	-	0.8
+1.20% MCC		320	429	34.2	-	-	1.2
+2.40% MCC		316	429	33.0	-	-	1.1
P.A.20:80	Air	299	426	39.5	554	3.7	0
+0.15% MCC		297	420	41.2	555	4.3	0
+0.30% MCC		298	421	39.6	550	4.3	0
+0.60% MCC		300	421	40.0	547	4.2	0
+1.20% MCC		298	419	41.2	547	4.1	0
+2.40% MCC		297	421	39.5	551	4.2	0

^a^ From derivative curve.

**Table 7:** Thermogravimetric data for CNC-based composites.

Sample Code	Atmosphere	T_5%_ (°C)	T_max1_^a^ (°C)	Residue at T_max1_ (%)	T_max2_^a^ (°C)	Residue at T_max2_ (%)	Residue at 700 °C (%)
P.A.20:80	Nitrogen	322	428	33.1	-	-	0.9
h		312	423	33.7	-	-	0.9
+0.30% CNC		315	424	35.8	-	-	1.0
+0.60% CNC		317	424	34.9	-	-	1.1
+1.20% CNC		316	424	34.4	-	-	1.2
+2.40% CNC		308	422	34.4	-	-	1.3
P.A.20:80	Air	299	426	39.5	554	3.7	0
+0.15% CNC		284	412	47.2	544	4.4	0
+0.30% CNC		290	417	45.0	544	4.5	0
+0.60% CNC		295	421	39.0	552	4.1	0
+1.20% CNC		292	420	40.3	550	4.2	0
+2.40% CNC		297	421	40.2	546	4.0	0

^a^ From derivative curve.

**Table 8:** Contact angle values with water for *PEGDA*:*AESO* 20:80 and its composites containing MCCs or CNCs. Statistical analyses were performed using Pearson’s correlation for MCC and CNC with *p* < 0.05.

Filler Type	Loading (wt. %)	Contact Angle (°)	Standard Deviation
-	-	93.0	2.3
MCC	0.15	61.9	5.1
	0.30	61.8	5.7
	0.60	60.5	3.8
	1.20	63.2	3.0
	2.40	59.0	4.0
CNC	0.15	51.9	2.1
	0.30	58.4	4.2
	0.60	53.9	0.7
	1.20	48.7	0.2
	2.40	52.1	2.4

**Table 9:** Depth penetration (*Dp*) and critical energy (*Ec*) as a function of MCC and CNC loadings. Statistical analyses were performed using Pearson’s correlation for MCC and CNC with *p* < 0.05.

Filler Type	Loading (wt. %)	*Ec* (mJ·mm^−2^)	*Dp* (mm)	*R* ^2^
MCC	0.15	0.94	0.34	0.98
	0.30	0.92	0.35	0.98
	0.60	1.06	0.35	0.96
	1.20	1.00	0.32	0.96
	2.40	0.97	0.32	0.97
CNC	0.15	1.16	0.39	0.99
	0.30	1.04	0.36	0.98
	0.60	1.05	0.37	0.96
	1.20	1.03	0.36	0.98
	2.40	1.04	0.35	0.97

## Data Availability

Not applicable.

## Supplementary Materials

The following supporting information can be downloaded at https://www.mdpi.com/article/10.3390/polym14091886/s1: Figure S1. FTIR spectra of MCC, P.A.20:80, and P.A.20:80 containing 2.4 wt.% MCC; Figure S2. FTIR spectra of CNC, P.A.20:80, and P.A.20:80 containing 2.4 wt.% CNC; Figure S3. Influence of different MCC mass percentages on the P.A.20:80 swelling after 5, 10, 20, and 30 days; Figure S4. Influence of different CNC mass percentages on the P.A.20:80 swelling after 5, 10, 20, and 30 days; Table S1. Contact angle measurements of 3D-printed samples with different *AESO* loadings.
