# Supplementary material for: Preparation and Characterization of 3D-Printed Biobased Composites Containing Micro- or Nanocrystalline Cellulose
Source: Polymers (Basel). 2022 May 5;14(9):1886. doi: 10.3390/polym14091886 (PMC9105471; doi:10.3390/polym14091886)
Supplement: Supplementary file 1 [file polymers-14-01886-s001.zip › polymers-1713507-supplementary.pdf]

polymers

Supplementary

Preparation and Characterization of 3D-Printed Biobased Composites Containing Micro- or Nanocrystalline Cellulose

Raphael Palucci Rosa [1,*], Giuseppe Rosace [2], Rossella Arrigo [3] and Giulio Malucelli [3]

1 Department of Engineering and Applied Sciences, University of Bergamo, Viale Marconi 5, 24044 Dalmine, Bergamo, Italy
2 Department of Engineering and Applied Sciences, University of Bergamo, Local INSTM Unit, Viale Marconi 5, 24044 Dalmine, Bergamo, Italy; giuseppe.rosace@unibg.it
3 Department of Applied Science and Technology, Politecnico di Torino, Local INSTM Unit, Viale T. Michel 5, 15121 Alessandria, Italy; rossella.arrigo@polito.it (R.A.); giulio.malucelli@polito.it (G.M.)
* Correspondence: raphael.rosa@unibg.it

Citation: Palucci Rosa, R.; Rosace, G.; Arrigo, R.; Malucelli, G. Preparation and Characterization of 3D-Printed Biobased Composites Containing Micro- or Nanocrystalline Cellulose. *Polymers* **2022**, *14*, 1886. https://doi.org/10.3390/polym14091886

Academic Editor: Andrea Ehrmann

Received: 20 April 2022
Accepted: 1 May 2022
Published: 5 May 2022

Publisher's Note: MDPI stays neutral with regard to jurisdictional claims in published maps and institutional affiliations.

Copyright: © 2022 by the authors. Submitted for possible open access publication under the terms and conditions of the Creative Commons Attribution (CC BY) license (https://creativecommons.org/licenses/by/4.0/).

1. FT-IR analysis

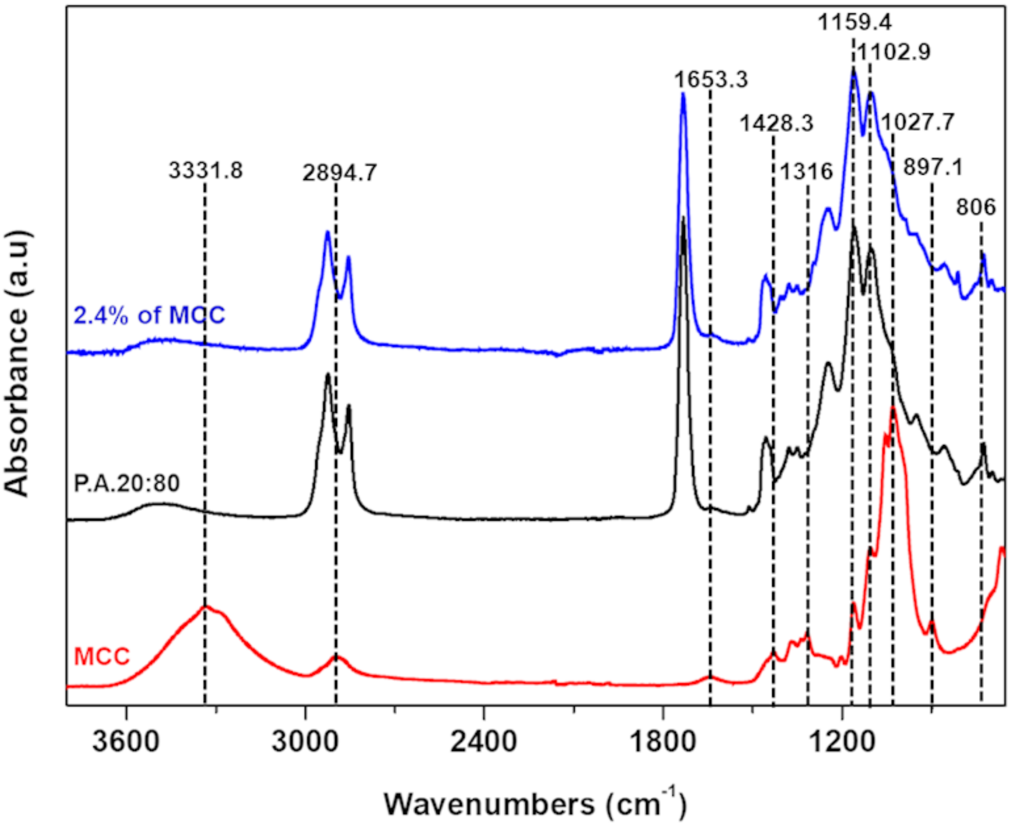

Figure S1: FT-IR spectra of MCC, P.A.20:80, and P.A.20:80 containing 2.4 wt.% of MCC.

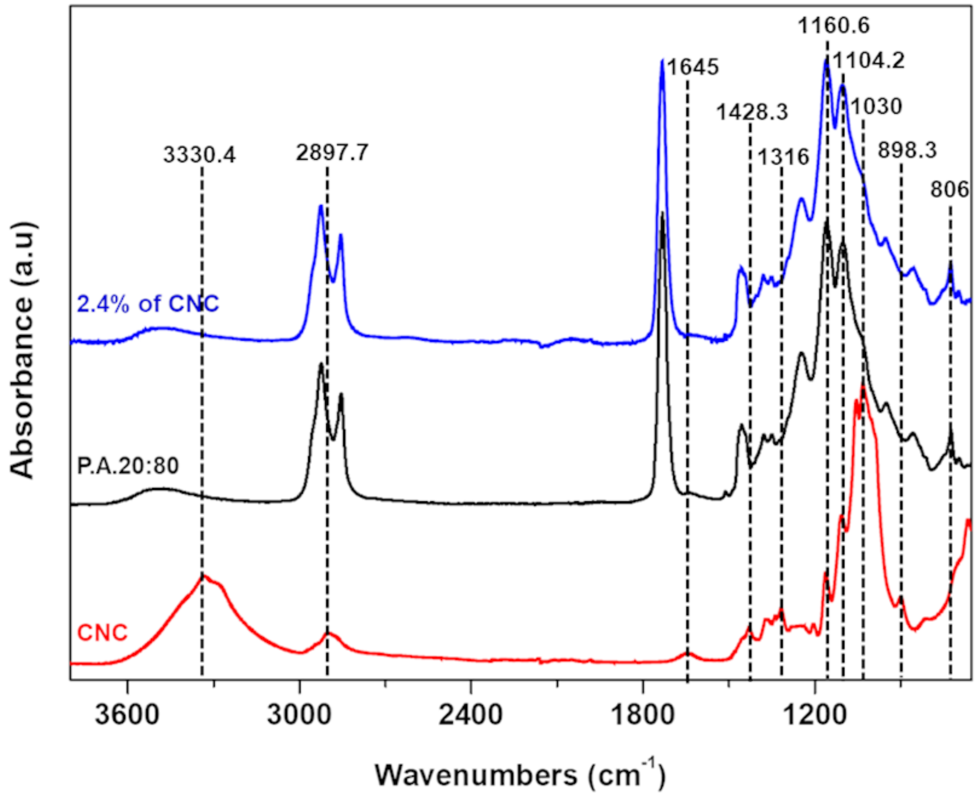

Figure S2: FT-IR spectra of CNC, P.A.20:80, and P.A.20:80 containing 2.4 wt.% of CNC.

2. Swelling properties

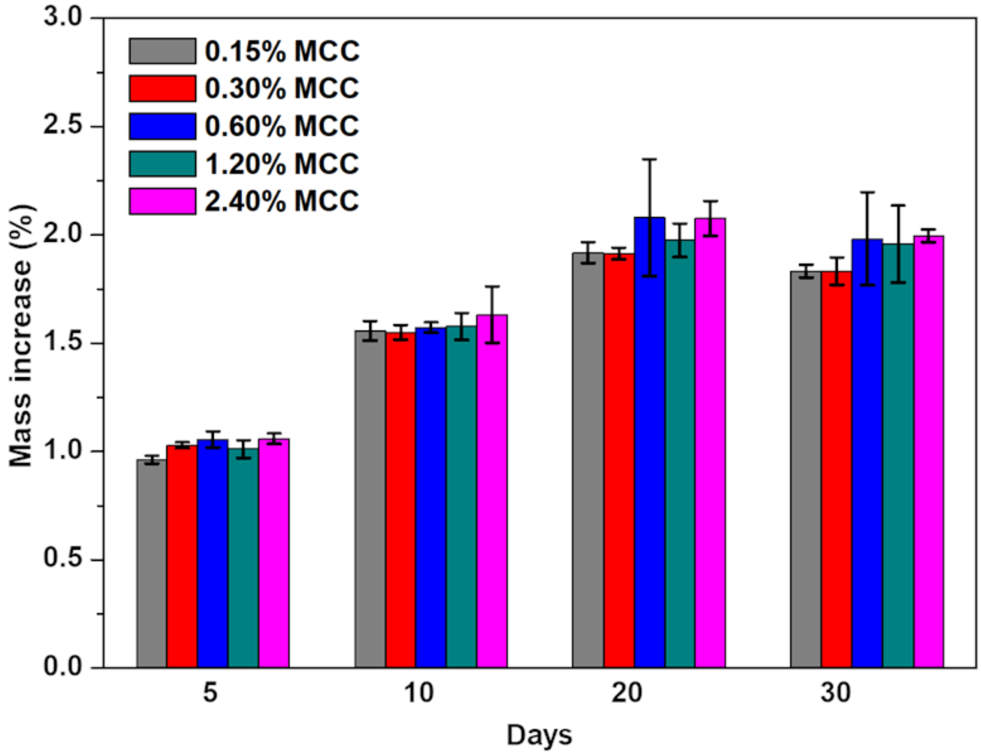

Figure S3: Influence of different MCC mass percentages on the P.A.20:80 swelling after 5, 10, 20 and 30 days.

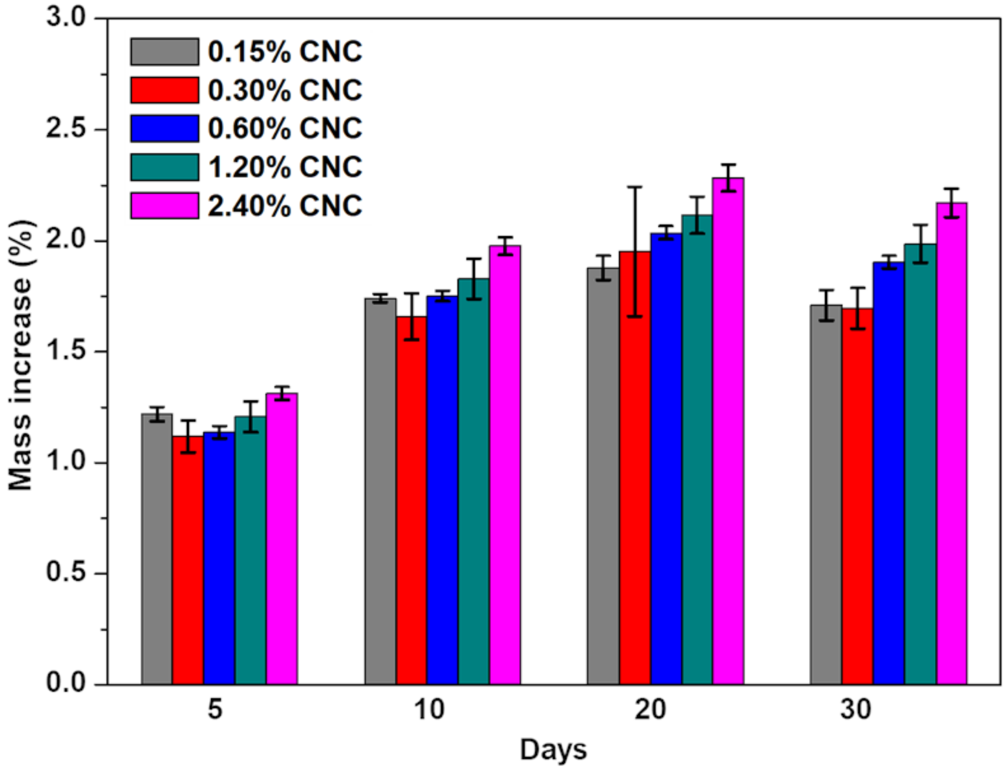

Figure S4. Influence of different CNC mass percentages on the P.A.20:80 swelling after 5, 10, 20 and 30 days.

3. Contact angle measurement

Table S1: Contact angle measurements of 3D-printed samples with different AESO loadings.

Sample code	Contact angle (º)	Standard deviation
PEGDA	72.1	3.8
P.A.50:50	76.8	2.7
P.A.40:60	78.5	0.8
P.A.30:70	88.6	0.9
P.A.20:80	93.0	2.3
AESO	92.0	1.6
